# Early and Late Effects of Cardiac Resynchronization Therapy in Adult Congenital Heart Disease

**DOI:** 10.1161/JAHA.119.012744

**Published:** 2019-10-28

**Authors:** Yanrong Yin, Konstantinos Dimopoulos, Eriko Shimada, Karen Lascelles, Samuel Griffiths, Tom Wong, Michael A. Gatzoulis, Sonya V. Babu‐Narayan, Wei Li

**Affiliations:** ^1^ Hospital of Xi'an Jiaotong University Xi'an China; ^2^ Royal Brompton Hospital Imperial College London London United Kingdom; ^3^ Department of Pediatric and Adult Congenital Cardiology Tokyo Women's Medical University Tokyo Japan

**Keywords:** adult congenital heart disease, cardiac resynchronization therapy, ventricular function, Congenital Heart Disease, Heart Failure

## Abstract

**Background:**

There are limited data about cardiac resynchronization therapy (CRT) in adult congenital heart disease. We aimed to assess early and late outcomes of CRT among patients with adult congenital heart disease.

**Methods and Results:**

We retrospectively studied 54 patients with adult congenital heart disease (median age, 46 years; range, 18–73 years; 74% men) who received CRT implantation (biventricular paced >90%) between 2004 and 2017. Clinical and echocardiographic data were analyzed at baseline and early (mean±SD: 1.8±0.8 years) and late (4.7±0.8 years) follow‐up after CRT. Compared with baseline, CRT was associated with significant improvement at early follow‐up in New York Heart Association functional class, QRS duration, and cardiothoracic ratio (*P*<0.05 for all); improvement in New York Heart Association class was sustained at late follow‐up. Among patients with a systemic left ventricle (LV; n=39), there was significant increase in LV ejection fraction and reduction in LV end‐systolic volume at early and late follow‐up (*P*<0.05 for both). For patients with a systemic right ventricle (n=15), there was a significant early but not late reduction in systemic right ventricular basal and longitudinal diameters. Eleven patients died, and 2 had heart transplantation unrelated to systemic ventricular morphological characteristics. Thirty‐five patients (65%) responded positively to CRT, but only baseline QRS duration was predictive of a positive response.

**Conclusions:**

CRT results in sustained improvement in functional class, systemic LV size, and function. Patients with a systemic LV and prolonged QRS duration, independent of QRS morphological characteristics, were most likely to respond to CRT.

**Clinical Perspective**

**What Is New?**

- This is the first study assessing both early and late impacts of cardiac resynchronization therapy in a relatively large population with adult congenital heart disease, reflecting real‐world contemporary, practical, and clinical response.
- The patients studied were the oldest with adult congenital heart disease in the literature, and the follow‐up is longer than currently documented in multicenter studies.
- Cardiac resynchronization therapy resulted in clinical improvement and reverse remodeling with a reduction in cardiac size and improvement in ventricular function; and baseline QRS duration, but not QRS morphological features, was the only significant predictor of cardiac resynchronization therapy response.

**What Are the Clinical Implications?**

- Cardiac resynchronization therapy should be considered in adult congenital heart disease, particularly in those with impaired systemic left ventricle and prolonged QRS duration.

## Introduction

Adult congenital heart disease (ACHD) is a rapidly expanding patient group. This nascent demographic phenomenon is creating major issues about the optimal management of patients with ACHD, in whom progressive heart failure is now the predominant mortality cause. Ventricular dyssynchrony appears to be common among these patients and can present at any stage of life. Cardiac resynchronization therapy (CRT) has been shown to improve exercise tolerance, heart failure symptoms, and survival in patients with left ventricular (LV) failure caused by idiopathic or ischemic dilated cardiomyopathy.^1^, ^2^ Recently, CRT has emerged as a potential treatment option in pediatric patients with congenital heart disease (CHD),^3^ but experience in ACHD remains limited in both patient numbers and follow‐up.^4^ We sought to assess the early and late effects of CRT in our patients with ACHD.

## Methods

### Data Availability Disclosure Statement

The authors declare that all supporting data and method descriptions are available within the article or from the corresponding author on reasonable request.

### Study Population

We retrospectively studied patients with ACHD and reduced systemic ventricular function (LV ejection fraction [LVEF] <40% or right ventricular fractional area change [RVFAC] <35%)^5^, ^6^ who received CRT in our tertiary ACHD center between 2004 and 2017 and had biventricular pacing at least 90% of the time. The study was approved by the local ethics committee, and the informed consent was waived.

### Device Implantation

At the time of study, there were no guidelines or robust consensus on indications for CRT specific to patients with ACHD. Decisions for device implantation in our study patients were made at multidisciplinary meetings, including ACHD cardiologists, electrophysiologists with interest in CHD, and CHD surgeons. In fact, we continue only making individualized device implantation decisions in this multidisciplinary format. In this retrospective series, indications may have evolved with time. For interest, in the context of the 2014 Pediatric and Congenital Electrophysiology Society/Heart Rhythm Society expert consensus statement on the recognition and management of arrhythmias in ACHD, in our study, there were 44 patients with systemic LVEF/RVFAC ≤35%, New York Heart Association (NYHA) functional class II to IV, and QRS ≥120 milliseconds; 3 patients with NYHA class I and >40% ventricular pacing; 5 patients with systemic LVEF >35% and >40% ventricular pacing; and 2 patients with systemic LVEF >35% and broadening QRS duration. The indications for CRT implantation were in the main compatible with more recently recommended practice.

Typically, when there was a bradycardia indication for pacing, the appropriateness of dual‐chamber versus biventricular pacing was routinely discussed at implantation or need for revision. The electrophysiologist in charge chose the appropriate commercially available device (Medtronic USA, n=29; Boston Scientific USA, n=15; St Jude Medical USA, n=10). After implantation, pacing parameters were optimized in all patients, as per routine clinical practice in our center.

### Follow‐Up

Data were retrieved from medical records and included demographics, cardiac diagnosis, surgical history, symptoms, and medication used; CRT system implantation, device‐related complications, heart transplantation listing, and death were all documented. NYHA functional class, ECG, cardiothoracic ratio, and echocardiography were analyzed at baseline, early (1–2 years after CRT) follow‐up, and late (4–5 years after CRT) follow‐up.

### ECG Data

Surface 12‐lead ECGs were acquired at a paper speed of 25 mm/s and a scale of 10 mm/mV. QRS duration was measured from its first deflection to its end. Complete left bundle branch block was defined as QRS duration ≥120 milliseconds, QS or rS form in lead V1, and broad R waves without Q waves in lead I and V6.^7^ Non–left bundle branch block included right bundle branch block, nonspecific intraventricular conduction delays, and predominantly paced rhythms with a nonphysiologic depolarization pattern.^8^

### Echocardiography

Standard M‐mode and 2‐dimensional echocardiographic views were used to assess LV end‐diastolic diameter and volume (LVEDD and LVEDV, respectively), end‐systolic diameter and volume (LVESD and LVESV, respectively), and LVEF by modified Simpson's method.^9^ Maximum transverse diameters at right ventricle (RV) basal, midlevel, and maximum longitudinal dimension were measured at end diastole. RV systolic function was assessed by measuring tricuspid annular plane systolic excursion and RVFAC, calculated as follows: [(end‐diastolic area–end‐systolic area)/end‐diastolic area]×100.^6^ Atrial volume index was calculated using the biplane area‐length formula, and left atrium and right atrium (RA) volumes were indexed to body surface area as left atrium/RA volume index.^10^

### Prespecified Definition of Positive CRT Response

Patients were considered responders to CRT if they exhibited ≥5% absolute increase in LVEF or RVFAC at echocardiographic follow‐up.^11^, ^12^, ^13^

### Statistical Analysis

Data analysis was performed using SPSS 19.0 for Windows (SPSS Inc, Chicago, IL). For all analyses, 2‐tailed *P*<0.05 was considered statistically significant. Continuous variables were presented as mean±SD or median (interquartile range), and categorical variables were expressed as count (percentage). For continuous variables with a normal distribution, paired and unpaired Student *t*‐test was used. For variables not normally distributed, the Wilcoxon signed rank test for paired samples and the Mann‐Whitney *U* test for independent samples were used. Matched categorical variables were analyzed using the McNemar test. Categorical variables for 2 independent groups were compared using the χ^2^ test or Fisher's exact test, according to sample size. For survival analysis, rates of survival were estimated using Kaplan‐Meier cumulative event curves. Univariate and multivariate stepwise logistic regression analysis was used to identify predictors of CRT response.

## Results

### Baseline Characteristics

Seventy patients with ACHD and reduced systemic ventricular function had received CRT implantation at our center between 2004 and 2017. Sixteen patients (22%) were subsequently excluded: 8 (11%) because of biventricular pacing was <90% of the time and 8 (11%) because of lack of available imaging data, leaving 54 patients (78%) included for study. Those who could not be studied because of lack of available echocardiography data were not different from those included in the study.

Baseline characteristics are listed in Table 1. Fifty‐four patients (mean age, 46±13 years; range, 18–73 years; 74% men) were followed up for a mean 5.7±3.0 years from CRT. Thirty‐nine patients (72%) had a systemic LV. Underlying cardiac anatomical features included LV outflow tract lesions (n=17; 32%), repaired tetralogy of Fallot (n=11; 20%), RV outflow tract lesions (n=5; 9%), atrioventricular septal defects (n=5; 9%), and atrial septal defect with right aortic arch (n=1; 2%). Fifteen patients (28%) had a systemic RV: 13 (24%) with congenitally corrected transposition of great arteries and 2 (4%) with transposition of the great arteries after Mustard repair. Three (6%) of the patients with congenitally corrected transposition of great arteries had not undergone previous surgery. Before CRT implantation, 33 patients (61%) had an indication for ventricular pacing because of a high degree atrioventricular block (n=30) or atrioventricular node ablation (n=3).

**Table 1:** Baseline Characteristics of Patients

Variable	Value (n=54)
Demographic and clinical characteristics	
Age at CRT implantation, y	46±13
Men	40 (74)
Follow‐up duration, y	5.7±3.0
SBP at CRT implantation, mmHg	112±15
DBP at CRT implantation, mmHg	70±10
BMI, kg/m^2^	25.7 (22.9–29.7)
Biochemical parameters	
Urea, mmol/L	7.1 (5.2–7.9)
Creatinine, μmol/L	84 (76–94)
ECG	
Sinus rhythm	44 (81)
Atrial fibrillation	10 (19)
QRS duration, ms	174±27
QRS morphological characteristics	
LBBB	15 (28)
Non‐LBBB	39 (72)
Device implantation	
PPM/ICD upgrade to CRT	31 (57)
CRT de novo	23 (43)
CRTD	46 (85)
CRTP	8 (15)
NYHA functional class	
I	3 (6)
II	20 (37)
III	28 (51)
IV	3 (6)
Drug treatment	
ACEI or ARB	52 (96)
β Blocker	48 (89)
Aldosterone antagonist	35 (65)
Loop diuretic	28 (52)
Amiodarone	11 (20)
Anticoagulation	36 (67)
Digoxin	5 (9)
Device‐related complications	
Infection	5 (9)
Lead dislodgement	3 (6)
Venous obstruction	1 (2)
Pneumohemothorax and pulmonary embolism	1 (2)

Values are mean±SD, median (interquartile range), or number (percentage). ACEI indicates angiotensin‐converting enzyme inhibitor; ARB, angiotensin receptor blocker; BMI, body mass index; CRT, cardiac resynchronization therapy; CRTD, CRT‐implantable cardioverter‐defibrillator; CRTP, CRT pacemaker; DBP, diastolic blood pressure; ICD, implantable cardioverter‐defibrillator; LBBB, left bundle branch block; NYHA, New York Heart Association; PPM, permanent pacemaker; SBP, systolic blood pressure.

### Device Implantation

Thirty‐one patients (57%) had a preexisting pacing system: 21 (38%) had a permanent pacemaker, whereas 10 (19%) an implantable cardioverter‐defibrillator. Forty‐six patients (85%) received a CRT‐implantable cardioverter‐defibrillator, whereas only 8 patients (15%) received a CRT pacemaker (Table 1).

In 52 patients (96%), resynchronization therapy involved implantation of a transvenous pacing lead into the RA and nonsystemic ventricle, whereas a systemic ventricular lead was placed transvenously into a coronary sinus branch. In 2 patients (4%), transvenous lead implantation was not feasible: in one patient, leads were placed via a combined transvenous and epicardial approach; the other patient received only epicardial pacing. Permanent atrial fibrillation was present in 10 patients (19%), and all of them had an RA lead positioned before CRT implantation. In 2 patients (4%), the RA lead was not connected to the generator; in 8 patients (15%), pacing programming was set to either VVIR (single chamber ventricular pacing and ventricular sensing with rate response) or DDIR (dual chamber atrial + ventricular pacing and atrial + ventricular sensing without atrial tracking but with rate response) mode.

There were 11 complications in 10 patients (19%) related to device implantation, with infection being the most frequent (n=5; 9%), followed by lead dislodgement (n=3; 6%) (Table 1). No differences in complication rates were observed between the systemic LV and systemic RV subgroups.

### Early and Late Effects of CRT in ACHD

**Figure 1:**
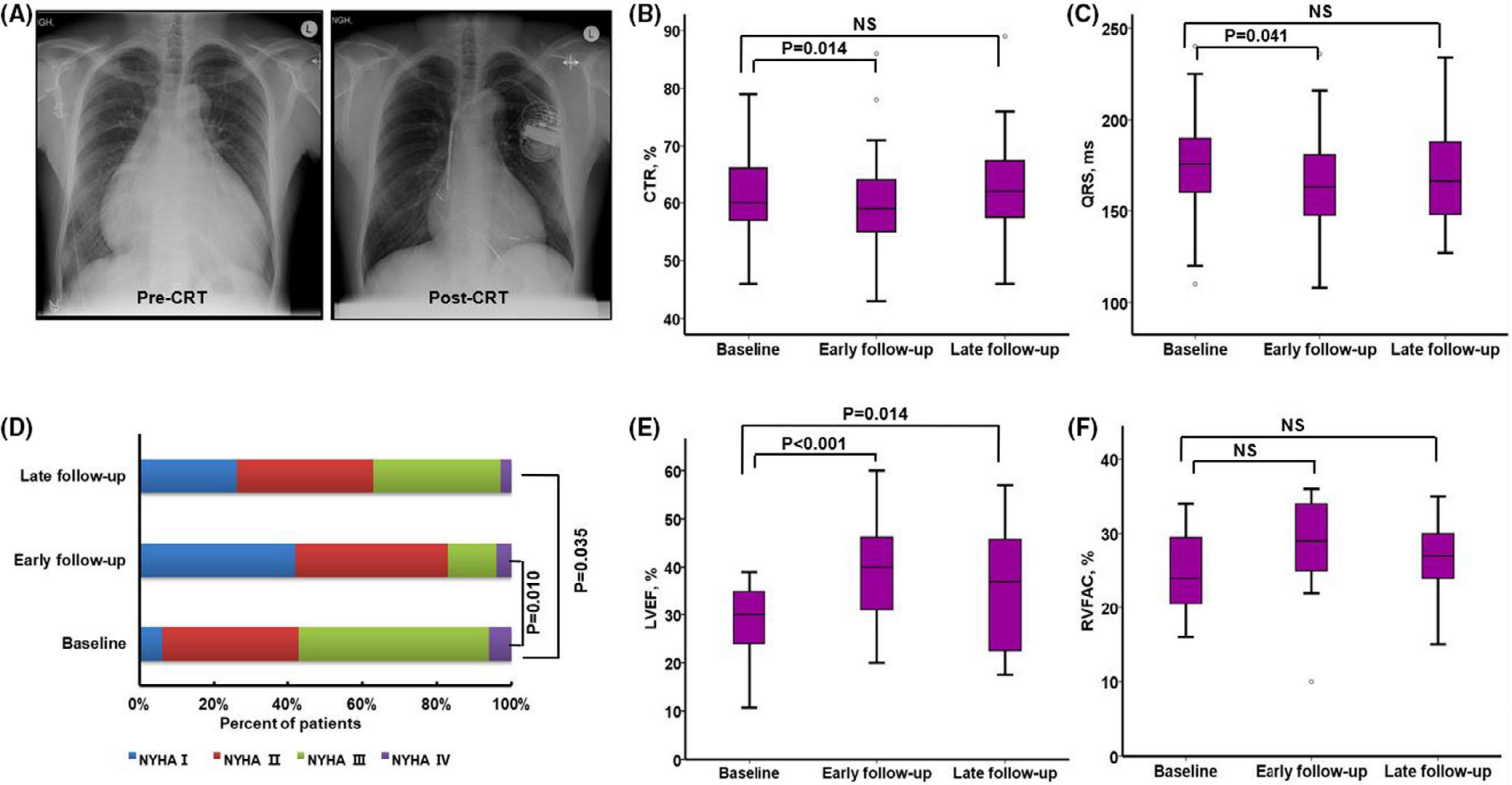
Illustration of changes pre–cardiac resynchronization therapy (CRT) and post‐CRT in patients with adult congenital heart disease. Example reduction of cardiothoracic ratio (CTR) of 0.79 preimplantation, which reduced to 0.55 at 1.2 years from CRT (**A**). There was a significant early, but not late, reduction in CTR (**B**) and QRS duration (**C**) in the overall population. An improvement in New York Heart Association (NYHA) functional class (**D**) was observed at early and late follow‐up in the overall population. There was a significant increase in left ventricular ejection fraction (LVEF) (**E**) at early and late follow‐up (*P*<0.05 for both) among patients with a systemic LV, whereas improvement of right ventricular fractional area change (RVFAC) (**F**) approached, but did not meet, statistical significance (*P*=0.070) among patients with a systemic RV. NS indicates not significant.

**Figure 2:**
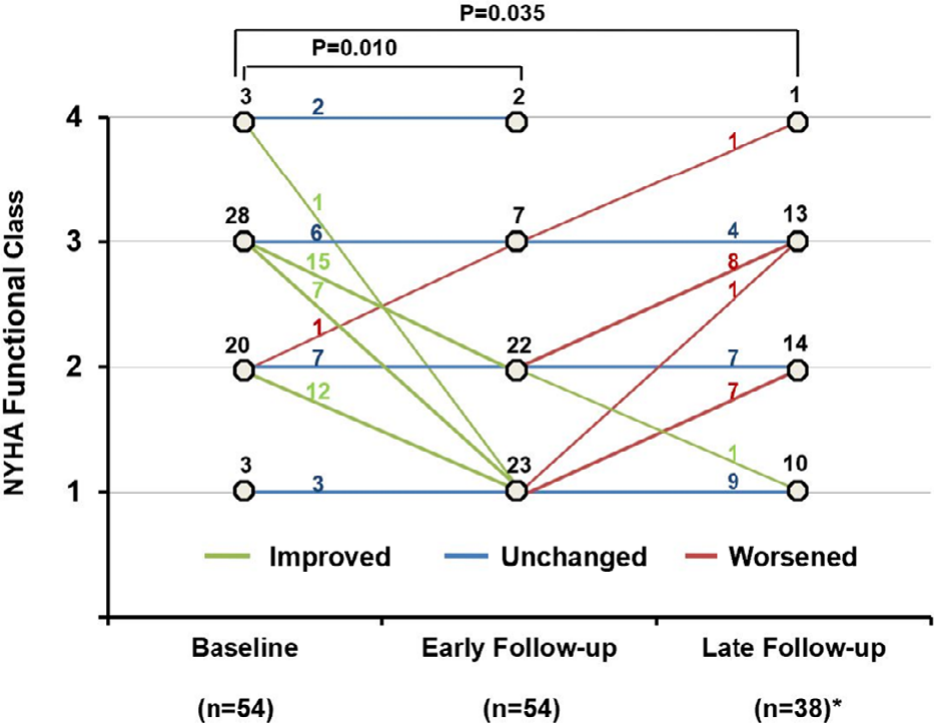
Change of New York Heart Association (NYHA) functional class pre–cardiac resynchronization therapy (CRT) and post‐CRT in an individual patient with adult congenital heart disease. *Sixteen patients had not reached the late follow‐up time.

The early effect of CRT in this population was assessed at a mean of 1.8±0.8 years, whereas the late effect was at 4.7±0.8 years after CRT implantation. Compared with baseline, CRT was associated with a significant improvement at early follow‐up in cardiothoracic ratio, QRS duration, and NYHA functional class (*P*<0.05 for all); only improvement in NYHA functional class was evident at late follow‐up (Figures 1 and 2).

**Figure 3:**
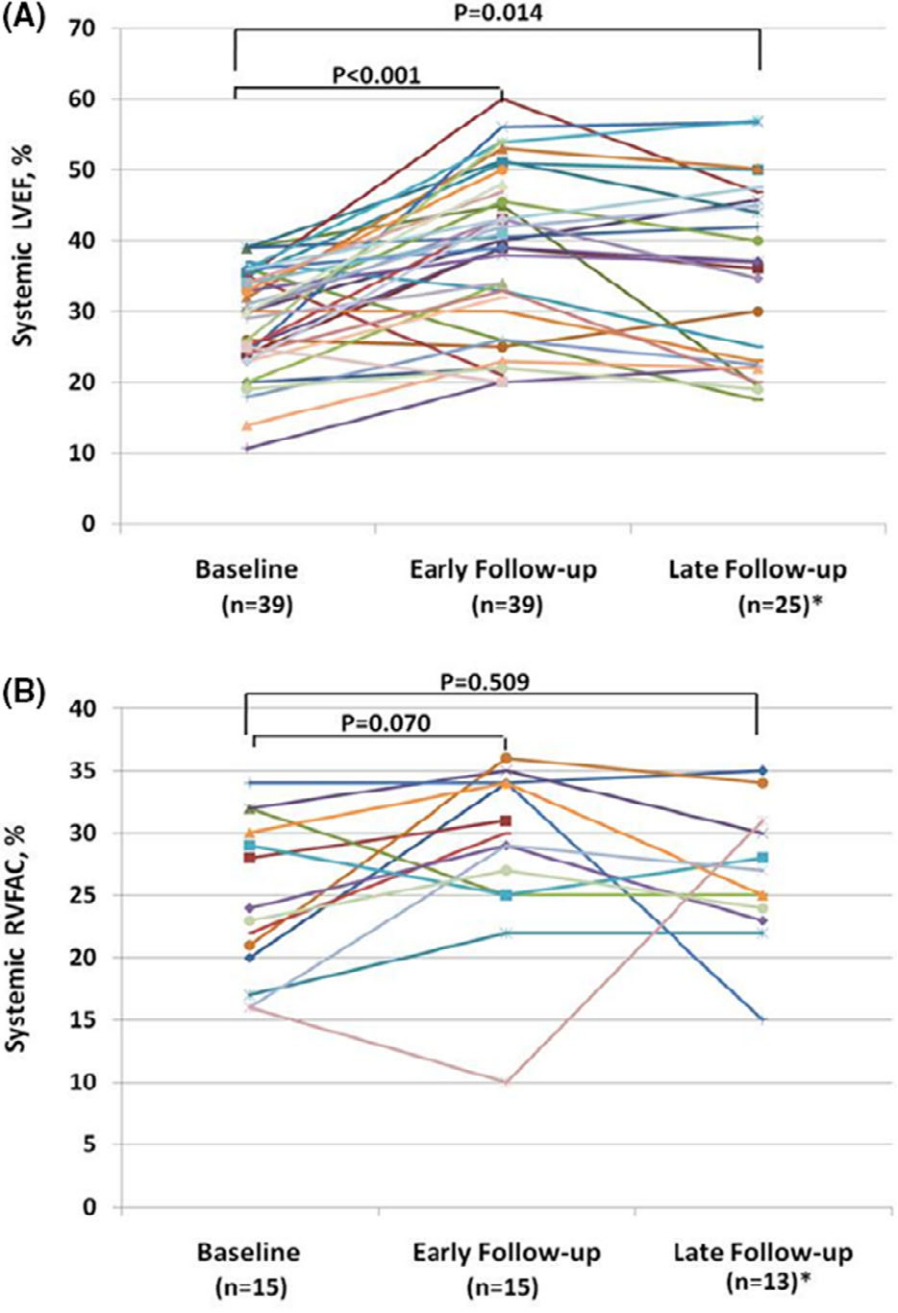
Changes of systemic left ventricular ejection fraction (LVEF) and right ventricular fractional area change (RVFAC) pre–cardiac resynchronization therapy (CRT) and post‐CRT in individual patient with adult congenital heart disease. *Fourteen patients with a systemic LV and 2 patients with a systemic RV had not reached the late follow‐up time.

In the 39 patients with a systemic LV (Table 2), significant improvement in LVEF and significant reduction in LVESV were observed at early and late follow‐up compared with baseline (*P*<0.05) (Figures 1 and 3A), whereas RA volume index, LVESD, and LVEDV were significantly reduced only at early follow‐up (*P*<0.05). In the 15 patients with a systemic RV (Table 3), there was a significant early, but not late, reduction in systemic RV basal and longitudinal diameters; improvement of RVFAC approached, but did not meet, statistical significance (*P*=0.070) (Figures 1 and 3B).

**Table 2:** Echocardiographic Measurements Early and Late From CRT in Patients With a Systemic LV

Variable	Pre‐CRT (n=39)	Early Follow‐Up (n=39)	*P* Value^a^	Late Follow‐Up (n=25)	*P* Value^b^
LAVI, mL/m^2^	47.0 (33.0–58.0)	40.0 (30.0–55.0)	0.081	37.0 (22.0–54.5)	0.363
RAVI, mL/m^2^	39.0 (26.0–55.0)	32.0 (22.0–51.0)	0.010	34.0 (26.5–62.0)	0.486
LVEF, %	28.9±7.1	38.6±11.2	<0.001	35.6±12.7	0.014
LVEDD, mm	63.2±10.2	60.7±9.7	0.052	60.3±11.5	0.111
LVESD, mm	51.7±10.5	48.1±11.6	0.043	48.0±13.4	0.190
LVEDV, mL	216±76	185±66	0.010	193±82	0.055
LVESV, mL	155±65	118±57	0.001	124±68	0.010

Values are mean±SD or median (interquartile range). CRT indicates cardiac resynchronization therapy; LAVI, left atrial volume index; LV, left ventricle; LVEDD, LV end‐diastolic diameter; LVEDV, LV end‐diastolic volume; LVEF, LV ejection fraction; LVESD, LV end‐systolic diameter; LVESV, LV end‐systolic volume; RAVI, right atrial volume index.

a Pre‐CRT vs early follow‐up.

b Pre‐CRT vs late follow‐up.

**Table 3:** Echocardiographic Measurements Early and Late From CRT in Patients With a Systemic RV

Variable	Pre‐CRT (n=15)	Early Follow‐Up (n=15)	*P* Value^a^	Late Follow‐Up (n=13)	*P* Value^b^
LAVI, mL/m^2^	31.0 (20.0–51.0)	34.0 (19.0–38.0)	0.274	42.0 (21.0–56.5)	0.275
RAVI, mL/m^2^	27.0 (23.0–35.0)	24.0 (19.0–35.0)	0.202	31.0 (16.5–42.5)	0.987
RVFAC, %	24.9±6.1	28.4±6.7	0.070	26.7±5.3	0.509
TAPSE, mm	12.0±3.2	12.2±2.9	0.842	10.8±2.4	0.265
RVD_basal_, mm	52.3±7.5	47.2±6.0	0.025	50.6±9.9	0.512
RVD_mid_, mm	55.5±12.1	50.5±8.7	0.110	54.9±11.4	0.819
RVD_longitudinal_, mm	75.1±9.2	71.3±9.2	0.026	71.1±11.5	0.216

Values are mean±SD or median (interquartile range). CRT indicates cardiac resynchronization therapy; LAVI, left atrial volume index; RAVI, right atrial volume index; RV, right ventricle; RVD, RV dimension; RVFAC, RV fractional area change; TAPSE, tricuspid annular plane systolic excursion.

a Pre‐CRT vs early follow‐up.

b Pre‐CRT vs late follow‐up.

**Figure 4:**
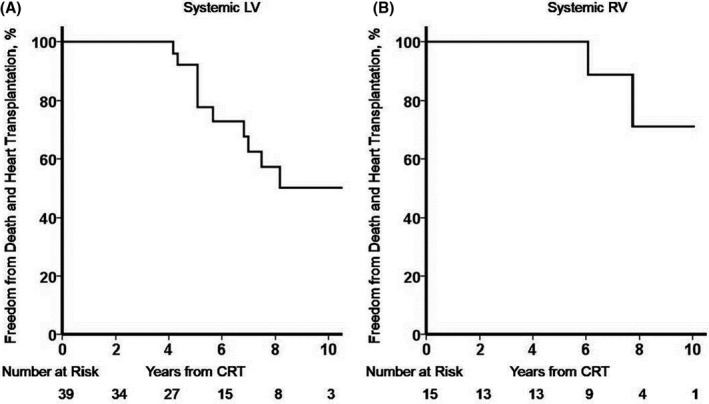
Survival freedom from death and heart transplantation. Kaplan‐Meier plots of patients with a systemic left ventricle (LV) (**A**) and a systemic right ventricle (RV) (**B**) after cardiac resynchronization therapy (CRT), with respect to freedom from death and heart transplant.

Eleven patients (20%) died from all‐cause mortality (range, 4.2–11.8 years after CRT). Five patients underwent CRT while listed for heart transplantation, 2 of whom had heart transplantation at 5 and 6 years from CRT, respectively; 2 improved sufficiently, allowing removal from the transplant waiting list; and 1 died. Figure 4 shows the Kaplan‐Meier curves depicting freedom from death and heart transplantation from CRT in patients with a systemic LV and RV.

### Predictors of Response to CRT

Among 54 patients, 35 (65%) were responders (≥5% absolute increase in LVEF or RVFAC). A favorable response to CRT was observed in most patients with a systemic LV (74%) versus less than half of patients with a systemic RV (40%). Within the systemic LV subgroup, the highest likelihood of response to CRT was manifested in LV outflow tract lesions, in which 15 of 17 (88%) responded. On univariate logistic regression analysis, the responders had a higher body mass index and a broader QRS duration, and more had a systemic LV compared with nonresponders, as summarized in Table 4. On multivariate stepwise logistic regression analysis, only baseline QRS duration was a significant independent predictor of response to CRT (odds ratio, 1.384 per 10‐millisecond increase of QRS duration; 95% CI, 1.042–1.838; *P*=0.025). There was no statistical difference in QRS duration between patients with a preexisting permanent pacemaker and those without (180±29 versus 171±26 milliseconds; *P*=0.240). Preexisting permanent pacemaker is not an independent predictor (odds ratio, 0.413; 95% CI, 0.131–1.301; *P*=0.131).

**Table 4:** Logistic Regression Analysis to Predict Response to CRT Among Baseline Parameters

Variable	Nonresponders (n=19)	Responders (n=35)	Univariate Analysis			Multivariate Analysis		
			OR	95% CI	*P* Value	OR	95% CI	*P* Value
Demography								
Age, y	41.6±14.3	48.2±12.4	1.040	0.994 to 1.088	0.087	…	…	…
Men	11 (58)	29 (83)	3.515	0.991 to 12.464	0.052	…	…	…
BMI, kg/m^2^	22.9 (21.7–27.1)	26.0 (24.2–31.6)	1.216	1.035 to 1.428	0.017	1.175	0.999 to 1.381	0.052
SBP, mm Hg	112±17	112±14	1.002	0.964 to 1.041	0.923	…	…	…
DBP, mm Hg	71±13	69±8	0.978	0.924 to 1.036	0.457	…	…	…
Systemic ventricle								
LV	10 (53)	29 (83)	4.350	1.236 to 15.312	0.022	3.470	0.832 to 14.470	0.088
RV	9 (47)	6 (17)						
NYHA function class								
I/II	5 (26)	18 (51)	0.952	0.901 to 1.006	0.080	…	…	…
III/IV	14 (74)	17 (49)	…	…	…	…	…	…
Laboratory variable								
Urea nitrogen, mmol/L	5.8 (5.0–7.5)	7.3 (5.6–8.6)	1.036	0.849 to 1.265	0.726	…	…	…
Creatinine, μmol/L	79.5±16.8	92.5±26.6	1.033	0.997 to 1.070	0.072	…	…	…
ECG								
Permanent AF	4 (21)	6 (17)	0.776	0.189 to 3.179	0.724	…	…	…
Sinus rhythm	15 (79)	29 (83)	…	…	…	…	…	…
QRS duration, ms^a^	159±29	182±23	1.449	1.105 to 1.900	0.007	1.384	1.042 to 1.838	0.025
QRS morphological characteristics								
LBBB	3 (16)	12 (34)	2.783	0.675 to 11.477	0.157	…	…	…
Non‐LBBB	16 (84)	23 (66)	…	…	…	…	…	…
Chest x‐ray								
CTR, %	59.5±6.8	63.2±7.4	1.081	0.989 to 1.181	0.086	…	…	…
CRT implantation								
Indication of V‐pacing	13 (68)	20 (57)	0.615	0.190 to 1.995	0.419	…	…	…
Preexisting PPM	10 (53)	11 (31)	0.413	0.131 to 1.301	0.131	…	…	…

Values are mean±SD, median (interquartile range), or number (percentage). AF indicates atrial fibrillation; BMI, body mass index; CRT, cardiac resynchronization therapy; CTR, cardiothoracic ratio; DBP, diastolic blood pressure; LBBB, left bundle branch block; LV, left ventricle; NYHA, New York Heart Association; OR, odds ratio; PPM, permanent pacemaker; RV, right ventricle; SBP, systolic blood pressure; V‐pacing, ventricular pacing.

a OR estimation is referred to per 10‐millisecond increase of QRS duration.

## Discussion

A significant improvement in cardiac remodeling and ventricular function was observed early after CRT in this ACHD intention‐to‐treat cohort. An improvement in functional class was observed at early and late follow‐up in the overall population, whereas the improvements in systemic ventricular size and function were sustained at late follow‐up only in patients with a systemic LV. Baseline QRS duration was the only significant predictor of CRT response in the total cohort. Our data support the use of CRT in ACHD, particularly in patients with left‐sided lesions, QRS prolongation, and an impaired systemic LV.

### Effects of CRT in ACHD

Although preliminary results in pediatric patients are encouraging,^3^, ^14^, ^15^, ^16^, ^17^ the application and outcomes of CRT in ACHD remain unclear. In our study, CRT was associated with a significant improvement in functional class, QRS duration, heart size, and LV function nearly 2 years after implantation. Only changes in NYHA class, LVEF, and LVESV were sustained at late follow‐up, perhaps reflecting the inevitable, progressive nature of symptomatic heart failure in ACHD.

Experience with CRT in patients with a systemic RV is limited. The largest study to date included 17 patients who received CRT, in whom there was a significant increase in systemic ventricular ejection fraction and decrease in QRS duration, with most (n=13; 76%) reporting a clinical improvement at a mean 4.8±4 months from CRT.^15^ In contrast, only 2 of 9 patients with a systemic RV and CRT from another study responded to therapy at ≈0.7 years from implantation.^14^ Our group recently reported that chronic subpulmonary LV pacing in patients with congenitally corrected transposition of great arteries results in clinical deterioration.^18^ Upgrading dual‐chamber pacing of subpulmonary LV to CRT may prevent the known long‐term deterioration often seen in this setting. Therefore, larger studies, in the form of prospective multicenter registries, are still warranted to confirm the effect of CRT in this unique patient group.

### QRS Duration and Response to CRT

Prolongation of QRS duration at baseline in our study was associated with positive response to CRT. Several studies in idiopathic or ischemic dilated cardiomyopathy have demonstrated that patients with longer QRS duration and left bundle branch block morphological characteristics have a greater response rate to CRT.^1^, ^19^ In CRT trials enrolling ACHD, most patients had antibradycardia pacing or non–left bundle branch block.^3^, ^4^, ^14^ Of note, our study patients had broad QRS duration and QRS morphological characteristics were not related to the response to CRT.

### Influence of Systemic Ventricular Morphological Characteristics on Response to CRT

A favorable response rate to CRT was observed in most patients with a systemic LV (74%) versus less than half of patients with a systemic RV (40%). Differences in the response to CRT between morphologically LVs and RVs are likely multifactorial, related to ventricular geometry and other factors (namely, myocardial architecture, hemodynamics, and electrical conduction patterns).^20^ Given the lack of robust guidelines particularly, with respect to patients with a systemic RV, an individualized multidisciplinary team approach is clearly required in decision making. Efficacy data of CRT in ACHD are derived mainly from 2 multicenter surveys,^3^, ^15^ 1 larger retrospective single‐center study,^14^ and several smaller case series. Most studies were retrospective, and follow‐up was largely limited to a few months.

Ours was the first study to assess both the early and late impacts of CRT in a larger ACHD population, reflecting real‐world contemporary practice and clinical response.

### Issues Related to CRT Implantation in ACHD

CRT for ACHD appears relatively safe when performed in a tertiary environment, although the complication rates in our patients with ACHD were higher than in general cardiology, in keeping with previous reports.^21^, ^22^, ^23^, ^24^, ^25^ Despite considerable challenges, however, technological innovations and better ACHD care have facilitated the application of CRT, even in patients with complex CHD.^26^ The vast majority of our patients had a device implanted using a transvenous approach, similar to clinical practice elsewhere,^24^ but 2 patients (4%) required an epicardial or hybrid approach. Despite the beneficial effects in selected patients with ACHD, as demonstrated in our study, the increased risk of device‐related complications should be kept in mind when considering counseling and consenting patients with ACHD for CRT.

### Limitations

This was a relatively small retrospective study, limiting the strength of the conclusions that may be drawn. Our population was heterogeneous and included a spectrum of different CHD lesions. The indications for CRT were not uniform, but rather individualized. Device programming was also not standardized. Quantitative functional assessments of 6‐minute walk distance, quality of life by the Minnesota score, and cardiopulmonary exercise testing were not available because of the retrospective nature of this study. Given the high cost of these devices, complexity of implantation in patients with ACHD, and the increased complication rate with additional hardware, we still do not have good guidelines to support widespread use. Larger studies, in the form of prospective multicenter registries, are required to establish the merits of CRT in patients with lesion‐specific ACHD and identify and validate selection criteria of patients who are most likely to benefit.

## Conclusions

CRT was beneficial in patients with ACHD selected through an individualized approach at a tertiary center and resulted in clinical improvement and reverse remodeling with a reduction in cardiac size and improvement in ventricular function. Patients with a systemic LV and prolonged QRS duration, independent of its morphological characteristics, were most likely to respond to CRT. Future studies in large, lesion‐specific ACHD cohorts are warranted to establish precise criteria for patient selection for CRT.

## Sources of Funding

Dr Babu‐Narayan was supported by the British Heart Foundation (FS/11/38/28864).

## Disclosures

None.
