# Early and Late Effects of Cardiac Resynchronization Therapy in Adult Congenital Heart Disease

**DOI:** 10.1161/JAHA.119.014507

**Published:** 2019-12-10

## Correction

In the article by Yin et al “Early and Late Effects of Cardiac Resynchronization Therapy in Adult Congenital Heart Disease,” which published on October 28, 2019, and appeared in the November 5, 2019 issue of the Journal (*J Am Heart Assoc*. 2019;8:e012744. DOI: 10.1161/JAHA.119.012744), corrections were needed.

Incorrect academic degrees were provided for authors Karen Lascelles, Samuel Griffiths, and Sonya V. Babu‐Narayan. Their degrees have been corrected as follows: Karen Lascelles, PGDip; Samuel Griffiths, MSc; Sonya V. Babu‐Narayan, MBBS, BSc, PhD.

The authors regret the errors.

The correction has been made to the current online version of the article, which is available here: https://www.ahajournals.org/doi/10.1161/JAHA.119.012744

